# Whole-to-parts causation mechanism

**DOI:** 10.3389/fpsyg.2026.1654139

**Published:** 2026-05-12

**Authors:** Yoshiyuki Ohmura, Yasuo Kuniyoshi

**Affiliations:** Department of Mechano-Informatics, Graduate School of Information Science and Technology, The University of Tokyo, Tokyo, Japan

**Keywords:** algebraic structure control, asymmetry between cause and effect, downward causation, inter-level causation, neural network model

## Abstract

How can the whole have a causal effect on its parts? This is considered impossible because, if the supervenient whole is completely determined by its parts, then the whole-to-parts causation would be redundant. However, the exclusion argument did not assume a hierarchy of multiple supervenient functions and the existence of an inter-level negative feedback control mechanism. Here, we propose that this mechanism enables a causal effect from the whole to its parts. Feedback control typically involves two mechanisms: observing and controlling of the feedback error. These mechanisms can be implemented at two different levels of the hierarchy. We assume that the macro-level consists of a set of mathematical functions that supervene on physical neural states. An algebraic structure of these functions describes a macro-level equation that determines the feedback error. This equation is independent of an external cause, introducing new causal power to the micro-level while avoiding overdetermination. Modifying the micro synaptic weights within the neural networks via inter-level negative feedback control is a whole-to-parts causation mechanism. It should be noted that this paper does not intend to take a position on the ontology surrounding downward causation.

## 1 Introduction

One of the key characteristics of consciousness and free will is that they originate within a system. In philosophy, the term “intrinsic” or “emergence” often refer to something generated within a system rather than through an input-output relationship. Although whole-to-parts causation has been discussed as a way to explain this phenomenon, the underlying mechanism remains unclear. This topic lies at the intersection of psychology and fundamental physics and is closely related to the mind-body problem.

British emergentism contended that wholes could have emergent properties not possessed by their parts. Inspired by British emergentism, (Sperry 1964, 1969, 1987) argued that macro-determinism saves volitional control and free will from epiphenomenalism. Sperry believed that downward causation (Campbell, 1977), which is the inter-level causation from the whole systems to their parts in the biological hierarchy, was promising mechanism for realizing macro-determinism (Sperry, 1987, 1991). However, the mechanism of inter-level causation from the whole to its parts remains unclear: how can the whole have a causal effect on its components? In this article, we propose that an inter-level feedback control mechanism enables downward causation from supervenient whole level to its parts level in the brain.

The causal framework assumed in this study is a mechanical account of causation (Salmon, 1985). The interventionist account of causation (Woodward, 2003), frequently employed in contemporary scientific research, is difficult to apply to the relationship between wholes and its parts without identifying causes at the whole level (Craver and Bechtel, 2007). This is because the method for changing the causes at the whole level independently of changes in the parts remains unclear. This study posits that causation holds when a cause at the whole level temporally precedes a change at the parts level.

Causes arising at the whole level must not alter outcomes at the parts level without a causal transmission mechanism. Furthermore, a causal transmission mechanism is necessary for causes arising at the whole level to trigger state changes at lower levels. In this study, we propose treating the inter-level feedback control mechanism as a causal transmission mechanism.

Traditional micro-determinism reduces all biological phenomena to physics and chemistry, and this reductionism assumes that all macro-level facts and laws can be deduced from micro-level laws and initial conditions. According to the micro-determinist view, whole-to-parts causation is impossible because the higher macro level is entirely determined by the lower micro level, rendering causation from whole to parts redundant (i.e., Kim, 2000's exclusion argument). Reductionists assume that conscious experience lacks causal power and is epiphenomenal. In contrast to the traditional view, Sperry's downward causation accepts that the whole system, which is physically composed of parts, has causal power over its parts.

(McLaughlin 1992) argued that downward causation from either the biological or chemical level, as claimed by British emergentists and Sperry, is incorrect. (Chalmers 2006) considered that downward causation was theoretically reasonable but that no examples of downward causation are known in the actual world. (Ellis 2009, 2012, 2019) gave the various examples of downward (top-down) causation, but for each given case, the examples are different from whole-to-parts causation we propose. Several debates exist on inter-level and macro-to-micro causation (Craver and Bechtel, 2007; Auletta et al., 2008; Hoel et al., 2013; Mossio et al., 2013; Bechtel, 2017; Noble et al., 2019; Rosas et al., 2020; Gontier, 2023). Some assumed that all interactions occur at the lowest level (Hoel et al., 2013; Craver and Bechtel, 2007; Bechtel, 2017). This is different from inter-level causation. (Craver and Bechtel 2007) argued that all of the interesting cases of “inter-level causation” are symmetrical: “components act as they do because of factors acting on mechanisms, and mechanisms act as they do because of the activities of their lower-level components.” However, actual causation must be asymmetrical (Pearl, 2000). Some argued that the macro-level can constrain micro-level by determining its initial and boundary conditions (Campbell, 1977; Auletta et al., 2008; Mossio et al., 2013; Noble et al., 2019). However, the connection between these macro-to-micro constraints and whole-to-parts causation remains an open issue^1^. To our knowledge, current literature on downward, cyclic, reticulate, or self-causation cannot explain the mechanism of whole-to-parts causation^2^.

According to (Bechtel 2008), there are reasons not to characterize the relation between a mechanism and its parts causally. Causes are often assumed to precede and be independent of their effects, in the sense that they are separate events. It is considered that supervenient macro cannot have independent causal power over the micro. Furthermore, the most problematic aspect of invoking causal vocabulary in inter-level causation is the issue of “overdetermination” (Bechtel, 2008). The same cause cannot determine an event redundantly from both the entire system and its parts.

If the supervenient macro is determined by the micro, how can the macro have a non-redundant causal effect on the micro? To address this question, we assume clarify a cause at the whole level and a causal transmission mechanism from whole level to parts level. In particular, this study assumes that causal transmission involves inter-level negative feedback control. To our knowledge, no one has ever examined whether downward causation is possible through inter-level negative feedback control. Negative feedback control involves two mechanisms: observing and controlling of the feedback error. Importantly, these mechanisms can be implemented at two different levels of the hierarchy. The macro is controllable because it is determined by the micro. However, the reverse is not true, indicating asymmetry. This is called inter-level negative feedback control.

Assuming there is inter-level negative feedback control, the preceding cause is an equation that defines the macro-level feedback error. Then, the micro-level components can be controlled to reduce the feedback error. If the macro-level equation is independent of the micro-level and external causes, then a change in the behavior of a component is caused by the macro-level feedback error. In this case, we can see that the control at the micro-level and the observation of feedback error at the macro-level are different events. Inter-level negative feedback control appears to avoid the “overdetermination” problem.

Here, we propose that inter-level feedback control using macro-level algebraic structural feedback error enables a causal effect from the whole to its parts. We assume that the macro-level in the brain consists of a set of mathematical functions. These functions supervene on physical neural states. These networks are composed of micro-level neurons that can control their synapses via negative feedback control. Changes in lower-level synapses may be caused by a higher-level feedback errors that cannot be explained by the lower level alone.

It is worth noting that, our formulation of whole-to-parts causation is not related to self-organization and emergence in dynamical systems or non-linear complex systems (Bedau, 1997; Gershenson et al., 2020). In non-linear complex systems, the state-to-state transition under the fixed micro mechanism is often the focus and negative feedback control is reduced to attractors. Without distinguishing between negative feedback control and attractors, it becomes impossible to formulate whole-to-parts causation we propose, since our concept relies on recognizing that observation and control occur at different hierarchical levels. We provide a model of whole-to-parts causation without emergence or self-organization.

The goal of typical negative feedback control is to converge a signal derived from measurement of the system output to a reference signal (equal to the desired output). The feedback error is the difference between the reference and the actual output signal. Given that this reference is often provided from outside the system, the reference is typically an external cause that does not depend on the micro inside the system. Such external causes are not suitable to define whole-to-parts causation, because the macro-level feedback error must be determined by the supervenient entities^3^. In this article, we argue that the equations used to define the algebraic structure of supervenient neural network modules can be used to determine macro-level feedback errors, which are independent of external causes.

## 2 A mechanism of whole-to-parts causation

Our whole-to-parts causation mechanism is based on three assumptions: a set of mathematical functions in the brain, negative feedback control at the micro-level, and macro-level feedback error based on algebraic structure of these functions.

### 2.1 Modular structure in the brain

(von Bertalanffy 1969) distinguished two biological hierarchies: structural and functional. An example of a biological structural hierarchy is the organization of the molecule, cell, tissue, organ, organism, population, and species. Meanwhile, a functional hierarchy is exemplified by the organization of the primary visual cortex, the secondary visual cortex, the association cortex, and the prefrontal cortex in the brain.

The biological structural hierarchy relates the whole to its parts. Since the whole is physically composed of its micro-level parts, no additional material is required. Such a structural hierarchy defines whole-to-parts causation. How the higher level acquires a non-redundant downward causal power is an open question, since this situation seems to be inconsistent with reductionism.

A functional hierarchy, on the other hand, assumes different physical variables, systems or agents at both the higher and lower levels, so that causation from the higher to the lower level is not whole-to-parts causation. In such cases, we refer to the non-supervenient macro as an external macro. Examples of the higher level system controlling the lower level system in the functional hierarchies are commonplace: a computer program in read-only memory controls a processor and memory outside the memory for the program, the central nervous system controls the peripheral nervous system, the motor cortex controls the muscle, and so on. The way to distinguish structural hierarchy from functional hierarchy is to note whether the physical entity is shared by higher and lower levels in the hierarchy (e.g., motor cortex and muscle are different physical entities in the functional hierarchy).

**Figure 1:**
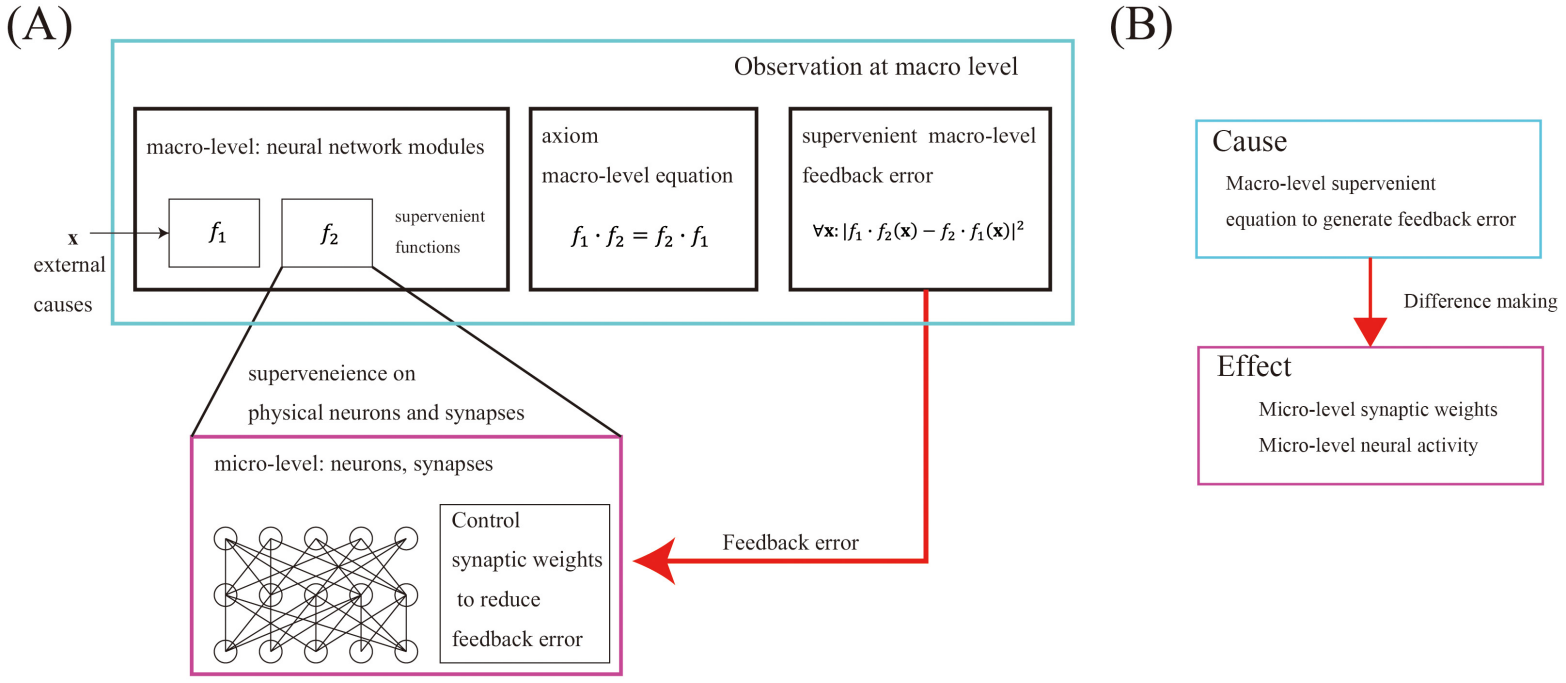
Whole-to-parts causation mechanism. **(A)** At the macro level, there are several neural network modules. Each neural network module consists of a set of neurons and synapses at the micro level. And the macro functions supervenes on the micro. An axiom is a macro-level equation used to calculate the supervenient feedback error. The axiom is definable at the macro level, independently of micro-states. At the micro level, synaptic weights within each neural network module are controlled to reduce the feedback error. Because the feedback error is described by macro-level functions independent of the external macro cause, we consider this mechanism as whole-to-parts causation. **(B)** We assume that a causal relation consists of a cause and a causal transmission mechanism. Changing the supervenient equation to calculate feedback error at the macro-level, the cause, affects the synaptic weights and neural activity at the micro level, the effect. The causal transmission is conducted by inter-level negative feedback control.

To formulate whole-to-parts causation, we assume a structural hierarchy in the brain. The brain is physically composed of neurons and several types of glial cells. For simplicity, we will focus only on neurons. A higher level organization must be physically composed of neurons and their synapses to share the physical entity between higher and lower levels in the hierarchy. Here, we assume the modular structure of neural networks at the macro level. Modular structures are most commonly found in the structural hierarchy of living organisms, and the neural network modules at the macro level are composed of a set of neurons and synapses (Figure 1A). The macro-level consists of a group of neural network modules, and the micro-level consists of the neurons and synapses within all the neural network modules, where the whole and its parts share the same physical entities. The columnar structures (Mountcastle, 1997), or memory engrams (Josselyn and Tonegawa, 2020), in the cerebral cortex suggests that there are multiple neural network modules in the brain. Since a neural network is often considered a mathematical function, we can assume that a set of mathematical functions, each function supervenes on the neurons and synapses within neural network modules of the brain.

This relationship between the whole and its parts is subsumed under the more general supervenience-subvenience relationship. Kim's Exclusion Argument (Kim, 2000) is often interpreted as denying hierarchical causation. However, Kim assumes that the higher-level hierarchy is a single supervenient entity and does not presuppose a hierarchy composed of multiple supervenient entities. Furthermore, while Kim assumes that the supervenient entity actively exerts influence over the subvenient entities, we assume that subvenient entities perform negative feedback control based on causes generated by the higher-level hierarchy. Therefore, our proposal lies outside the scope of Kim's argument.

Furthermore, although this study does not address the issue, Kim adopts a philosophical stance that does not distinguish between physical determinism and physical causal closure; this view differs from the currently dominant theories of causation. Since it is now generally accepted that physics does not define the concept of causation, physical causal closure and physical determinism should not be equated. Thus, Kim's argument contains many points that require reconsideration from a contemporary perspective.

### 2.2 Negative feedback control at the micro-level

(Kim 2006) argued that there is no causal power at the macro level because macro-level causality implies “overdetermination”; therefore, the whole structure cannot exert non-redundant causal power over the micro parts. This “causal exclusion” argument is often applied to argue against the possibility of mental causation beyond physical causation (Kim, 2000), but it can be applied to all cases of supervenience, including the hierarchy of sciences (Bontly, 2002). According to (Bontly 2002), Kim's position actually implies that only the properties of fundamental physical particles at the micro level are causally effective. To support whole-to-parts causation, we must reject Kim's argument.

(Kim 2006) assumed that higher level properties (corresponding to the macro) must directly cause the lower base properties (corresponding to the micro), without negative feedback control. However, we consider that the micro-level neurons actively control synaptic weights to reduce supervenient macro-level feedback error, rather than being passively controlled or superseded by the supervenient macro. This mechanism avoids “overdetermination” while maintaining lower basic laws.

We assume that the micro-level consists of synaptic weights, and the macro-level consists of mathematical functions (i.e., neural networks) that supervene on the physical neurons and synaptic weights. An equation to define feedback error is described by the macro-level functions, and this equation cannot be described at the micro-level alone. The equation for supervenient feedback error can be determined at the macro level independently of micro-physics, suggesting that inter-level negative feedback control supports macro-determinism. The underlying cause at the higher level is the modification of the equation used to calculate the feedback error through the selection and rearrangement of multiple supervenient functions. This modification can be performed independently of state changes at the micro-level. Kim did not assume that the hierarchy was composed of such multiple supervenient entities. Importantly, our whole-to-parts causation is asymmetry. Causal transmission via inter-level negative feedback control is possible only in the direction from the macro level to the micro level.

To explain the macro-determinism, the macro-level equation for feedback error must change dynamically. The macro-level mechanism that governs this change is independent of micro-physical laws, yet it is embodied in the brain. Additional neural circuits outside of the whole-parts hierarchy are necessary to change the equation for inter-level negative feedback error. Assuming an external mechanism to alter equations for feedback error risks falling into an infinite regress. However, we believe the situation differs because, unlike considering causality horizontally within the same hierarchy, the whole-parts relationship cannot be defined infinitely in the vertical direction due to coarse-graining. We expect this mechanism to use mathematics and logic to generate structured perception, intelligence, and adaptive behavior that evolve in response to the environment. Interestingly, macro-determinism acknowledges that there are different macro-level dynamics among individuals. However, this article does not address the principle by which the equation is autonomously determined. Future work must clarify the relationship between the higher-level causes and the functional development of perception and adaptive behavior. This will allow us to develop a theory of the macro-level mechanism that governs changes in the higher-level causes.

### 2.3 Macro-level feedback error based on algebraic equation of the macro-level functions

We assume that the brain has multiple neural network modules at the macro level. Each neural network module is a mathematical function or map with plasticity. A mathematical property of the functions supervenes on the synaptic weights.

Note that a macro-level function “alone” cannot regulate the synaptic weights that make up the function itself due to supervenience. Macro level feedback error cannot be defined by any single neural network module because only the identity equation *f* = *f* can be defined by the function *f* and = alone. Therefore, we assume a macro-level equation is described by multiple macro-level functions and algebraic operations (Figure 1A). We assume that the algebraic operations are static and not causal difference makers.

An algebraic structure is defined by a set *S* with operation “·” in mathematics. We assume that a set is composed of the macro-level functions labeled by lowercase letters { *f*_1_, *f*_2_, *f*_3_ ... } . A binary operation combines any two elements *f*_1_ and *f*_2_ of *S* to form an element of *S*, denoted *f*_1_·*f*_2_. We assume that a binary operation “·” is a function composition between macro-level functions.

An algebraic structure satisfies several requirements, known as axioms. The axioms are usually described in terms of equations in group theory. To define such axioms, the function composition operation is required between the neural network modules. To satisfy this condition, the neural network modules must be transformations whose inputs and outputs are elements of a set such as vectors with the same dimension. We assume that an input and output for the neural networks are elements of a set and labeled by bold letters { **x**, **y** ... }. Here, the input and output **x**∈ℝ^*n*^ are external causes and do not supervene on the micro-level neurons and synaptic weights within the system.

The axioms are equations defined by a set of macro-level functions and the binary operations. The axioms for the algebraic structure can only be defined at the macro level because the modules are defined at the macro level structure. Furthermore, if the axioms are satisfied in all inputs and outputs, then the axioms can be described by the macro-level functions alone, indicating the independence from the external causes. These features (supervenience, and independence from external causes) are required for the macro-level equation to explain whole-to-parts causation by inter-level negative feedback control.

Macro-level feedback error can be expressed as follows: ∀**x**:*E*_*macro*_(*f*_0_, *f*_1_, ...)(**x**).

Here, *E*_*macro*_ are derived from equations consisting of macro-level functions and the binary operation. And *E*_*macro*_(*f*_0_, *f*_1_, ...) is a function whose domain and codomain are ℝ^*n*^.

In this article, we argue that the brain system can evaluate an algebraic structural feedback error described by the macro-level functions to regulate synaptic weights at micro level.

When the synaptic weights are denoted as *W*, the synaptic weights are changed according to the following equation: Δ*W* = −η*∂E*_*macro*_(*f*_0_, *f*_1_, ...)(**x**)/∂*W*. Here, η is a feedback gain.

### 2.4 An example mechanism of whole-to-parts causation

In this section, we propose that an equation defining the algebraic structure between neural network modules can be used to measure supervenient feedback error at the macro level. We assume that the neural network module is mathematically modeled as a function. And algebraic axioms is described by the neural network modules and algebraic operation. The algebraic structural feedback error to satisfy the algebraic axioms can be used for inter-level negative feedback control from the macro level to the micro level.

In mathematics, axioms are prerequisites, but we think of axioms as control targets that constrain the algebraic structure of the macro-level functions through negative feedback control. The algebraic structure of the macro-level functions, without negative feedback control, is not limited to the mathematical structures we commonly know. For example, commutativity is not satisfied by all algebraic structures. Commutativity is satisfied by products of integers and real numbers, but not by matrices. If commutativity holds between neural networks, it is because the brain has developed to satisfy the axiom through negative feedback control.

In the brain, the axioms between neural network modules that are the target for feedback control can only be defined at the macro level. Therefore, a feedback error can only be evaluated at the macro level. Regulating its synaptic weights to reduce the feedback error is all the component neuron mechanisms do at micro level. The master controller to determine the target axioms and feedback error is derived at the macro level. Changing in the supervenient equation to calculate feedback error at the macro-level (the cause) affects the synaptic weights and neural activity at the micro level (the effect) (Figure 1B). This mechanism enables macro-determination. We call this asymmetric mechanism algebraic structure control.

In machine learning, these synaptic weights changes are enabled by gradient descent such as error back propagation (Rumelhart et al., 1986). For example, consider commutativity axiom as a feedback error to satisfy a target algebraic structure and assume that **x** is input to two neural network modules, *f*_1_ and *f*_2_. The feedback error is defined as $\forall \text{x}:{\left(f_{1}\cdot f_{2}\left(\text{x}\right)-f_{2}\cdot f_{1}\left(\text{x}\right)\right)}^{2}$ (Ohmura et al., 2023; Nishitsunoi et al., 2024). To satisfy commutativity (which is not generally satisfied), the synaptic weights must be adjusted at the micro level. In this situation, the supervenient feedback error about the commutativity between neural network modules, *f*_1_ and *f*_2_, can be used to regulate the synaptic weights within the modules themselves at the micro level. This whole-to-parts causation mechanism is logically plausible in the brain.

**Figure 2:**
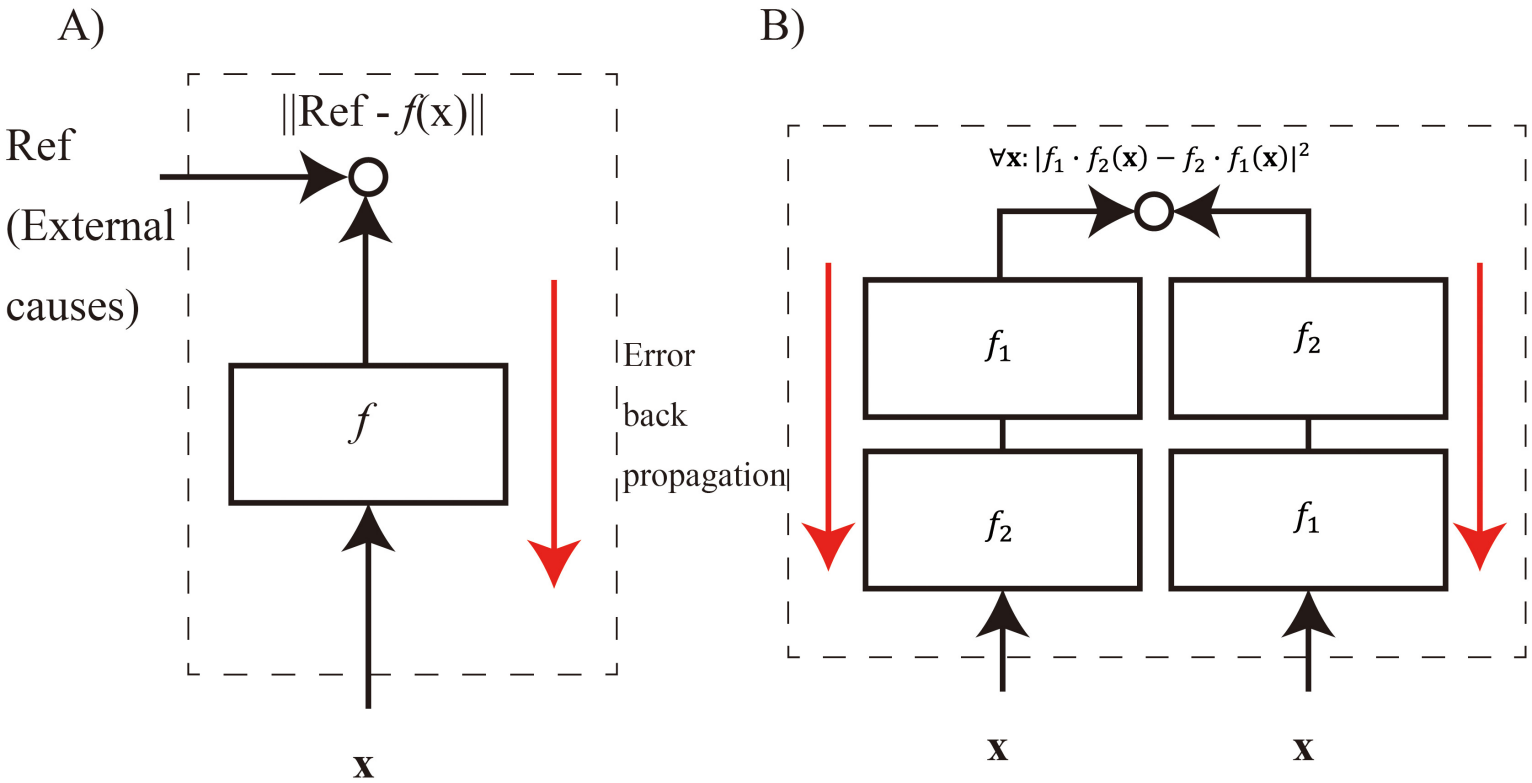
The difference between macro-to-micro causation and whole-to-parts causation. Both update micro-level synaptic weights using macro-level description. However, defining supervenient feedback error using an external reference is problematic, since the relationship between the reference and the micro is not clear. **(A)** In macro-to-micro causation, the feedback error is defined using a reference signal from the outside of the system. **(B)** In whole-to-parts causation, the feedback error is defined by axioms to satisfy a target algebraic structure such as commutativity between the macro-level functions, *f*_1_ and *f*_2_.

In conventional deep learning, a type of macro-to-micro causation, the feedback error to control the synaptic weights depend on an external macro cause, including a reference signal supplied from the outside of the system (Figure 2A). In this case, the non-supervenient external macro is the cause of the synaptic weight changes at the micro level. In supervised learning, the labels of the data are given as a reference. In predictive learning, the data at different times are used as a reference. In unsupervised learning, this is often done to approximate the data distribution to a probability distribution, such as a Gaussian distribution. The target and input X do not supervene on the micro. Therefore, the error between X and target is not suitable to define whole-to-parts causation.

A unique feature of algebraic structure control is that the feedback error is defined by the macro-level functions independent of an external macro (Figure 2B). Independent of external cause means that the algebraic equation defined at the macro level is not determined by external causes or micro-states. This feature makes it possible to generate a cause that can be intervened within the system at the macro level. Additionally the feedback error can be evaluated within the system without the need for external reference signals. Therefore, we consider that algebraic structure control is to be a model of whole-to-parts causation.

## 3 Proof of concept

We implement an example of whole-to-parts causation mechanism. We used error back propagation (Rumelhart et al., 1986) to control synaptic weights. We show that commutativity at the macro level can be controlled by the synaptic weights at the micro level.

We define a neural network that learns to convert input **x** to output **y**. Without a feedback error at the macro level, the loss function is described by ||**y**−*f*[**λ**](**x**)||. To realize commutativity at the macro level, additional commutative loss, ||*f*_1_[**λ**_1_]·*f*_2_[**λ**_2_](**x**)−*f*_2_[**λ**_2_]·*f*_1_[**λ**_1_](**x**)|| is required. Here, **λ**, **λ**_1_, **λ**_2_ are vectors determined from **x** and **y**.

We assume that the function *f*[**λ**] transforms from the whole input features **x** to the output features **y** for the conversion. The functions *f*_1_[**λ**_1_], *f*_2_[**λ**_2_] replace a part of the features and a combination of both *f*_1_ and *f*_2_ (i.e., *f*_1_[**λ**_1_]·*f*_2_[**λ**_2_] and *f*_2_[**λ**_2_]·*f*_1_[**λ**_1_]) replace all the features (Figure 2B). To realize this, **x** is encoded into two latent vectors *x*_1_ and *x*_2_ (Equation 1). **y** is also encoded into two latent vectors *y*_1_ and *y*_2_ (Equation 2).


$$\begin{array}{l} x_{1},x_{2}=G_{e}\left(\text{x}\right) \end{array}$$ (1)

$$\begin{array}{l} y_{1},y_{2}=G_{e}\left(\text{y}\right) \end{array}$$ (2)

The function *f*[**λ**] replaces both *x*_1_ and *x*_2_ to *y*_1_ and *y*_2_. On the other hand, *f*_1_[**λ**_1_] replaces only *x*_1_ to *y*_1_, where **λ**_1_ = *y*_1_−*x*_1_. Similarly, *f*_2_[**λ**_2_] replaces only *x*_2_ to *y*_2_, where **λ**_2_ = *y*_2_−*x*_2_. The output **y**, *f*_1_[**λ**_1_](**x**), and *f*_2_[**λ**_2_](**x**) are decoded from the latent vectors (Equations 3–5).


$$\begin{array}{l} \text{y}=f\left[\lambda \right]\left(\text{x}\right)=G_{d}\left(y_{1},y_{2}\right) \end{array}$$ (3)

$$\begin{array}{l} f_{1}\left[\lambda_{1}\right]\left(\text{x}\right)=G_{d}\left(y_{1},x_{2}\right) \end{array}$$ (4)

$$\begin{array}{l} f_{2}\left[\lambda_{2}\right]\left(\text{x}\right)=G_{d}\left(x_{1},y_{2}\right) \end{array}$$ (5)

*f*_1_[**λ**_1_]·*f*_2_[**λ**_2_](**x**) and *f*_2_[**λ**_2_]·*f*_1_[**λ**_1_](**x**) can be obtained using the same encoder and decoder (Equation 6–9).


$$y_{1}^{'},x_{2}^{'}=G_{e}(f_{1}[\lambda_{1}](x))$$ (6)

$$f_{2}[\lambda_{2}]\cdot f_{1}[\lambda_{1}](x)=G_{d}(y_{1}^{'},y_{2})$$ (7)

$$x_{1}^{'},y_{2}^{'}=G_{e}(f_{2}[\lambda_{2}](x))$$ (8)

$$f_{1}[\lambda_{1}]\cdot f_{2}[\lambda_{2}](x)=G_{d}(y_{1},y_{2}^{'})$$ (9)

The overall objective is to minimize both the reconstruction error and commutativity error between *f*_1_ and *f*_2_ (Equation 10). Encoder *G*_*e*_ and decoder *G*_*d*_ are neural networks and the synaptic weights are trained by loss function (Equation 11):


$$\begin{array}{l} loss_{c}=\|f_{1}[\lambda_{1}]\cdot f_{2}[\lambda_{2}](x)-f_{2}[\lambda_{2}]\cdot f_{1}[\lambda_{1}](x)\|\;\\ \;=\|G_{d}(y_{1}^{'},y_{2})-G_{d}(y_{1},y_{2}^{'})\| \end{array}$$ (10)

$$\begin{array}{l} loss=||\text{y}-G_{d}\left(y_{1},y_{2}\right)||+gain*loss_{c}, \end{array}$$ (11)

where *loss*_*c*_ is a feedback error at the macro level to satisfy commutativity between *f*_1_ and *f*_2_ (Figure 2B). In whole-to-parts causation condition, we set *gain* to 1. In the ablation condition, we set *gain* to 0.

### 3.1 Method

#### 3.1.1 Dataset

The dataset consists of 26 alphabets with 12 fonts and seven colors. The image size is three channels × 32 pixels × 32 pixels. The background color is black, (0,0,0) in (R, G, B). The seven colors of alphabets employed consist of (0, 0, 1), (0, 1, 0),…, (1, 1, 1). Two images are randomly sampled to make pairs of (**x**, **y**). To increase the variety of colors, a random value from 0.2 to 1 was multiplied to the color channels.

#### 3.1.2 Encoder *G*_*e*_

Two latent vectors are encoded by two different encoders *G*_*e*1_ and *G*_*e*2_.


$$\begin{array}{l} x_{1}=G_{e1}\left(\text{x}\right) \end{array}$$ (12)

$$\begin{array}{l} x_{2}=G_{e2}\left(\text{x}\right) \end{array}$$ (13)

$$\begin{array}{l} y_{1}=G_{e1}\left(\text{y}\right) \end{array}$$ (14)

$$\begin{array}{l} y_{2}=G_{e2}\left(\text{y}\right) \end{array}$$ (15)

*G*_*e*1_ (Equations 12, 14) and *G*_*e*2_ (Equations 13, 15) are different neural networks with different synaptic weights but the network structures are the same. The input image is convolved using three Convolutional Neural Networks (CNN) (Krizhevsky et al., 2012) whose kernel size is 4, stride is 2 and padding is 1 without bias, and two linear layers follow. We used ReLU function for activation. The channels of the convolution layers were 128, 256 and 512. The output dimensions of the linear layers were 16 and 32. The number of dimension of the latent vectors was 32.

#### 3.1.3 Decoder *G*_*d*_

The input was a tuple of two latent vectors. The network consisted successively of three linear layers without bias and three transposed convolution layers whose kernel size is 4, stride is 2, and padding is 1 without bias, and final layer is Conv1 × 1 layer (Lin et al., 2014). The output channels of the linear layers were 128, 1,024, and 4,096. The output channels of the transposed convolution layers were 128, 64, and 32. We used the same network configuration using different initializations for comparison.

#### 3.1.4 Training

We used RAdam (Liu et al., 2020) for optimization. The learning rate was 1e-4 and batch size was 128. We used CUDA 11.4, PyTorch 1.10.0 (Paszke et al., 2019) and Nvidia RTX 3080Ti (NVIDIA Corporation, Santa Clara, USA) for training. Training epochs were 400. All program codes are available at https://github.com/Yoshiyuki-Ohmura/DownwardCausation.

### 3.2 Result

**Figure 3:**
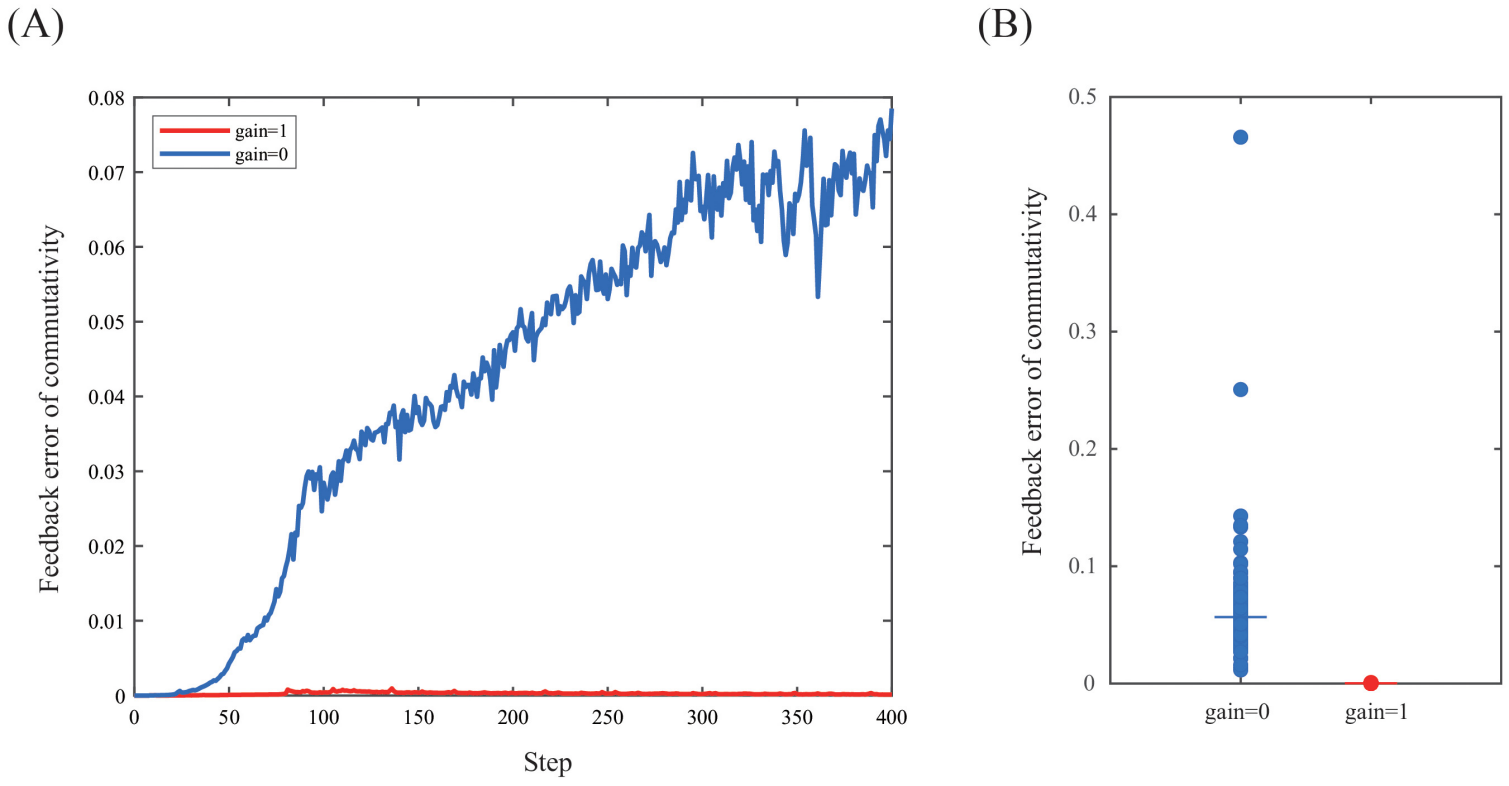
Feedback error of commutativity loss (*loss*_*c*_). **(A)** When feedback gain is 0, the feedback error increased. In contrast, when feedback gain is 1, the feedback error converged to 0. Horizontal axis shows time step. **(B)** Feedback error of commutativity is significantly different between feedback control condition (*gain* = 1) and ablation condition (*gain*= 0) (Mann–Whitney *U* = 10,000, *n* = 100, *p* < 2.5e-4, two-tailed) The median of feedback error in feedback control condition is 1.76e-4. The median of feedback error in ablation condition is 0.056.

When feedback gain of the commutativity is 0, the synaptic weights inside *G*_*e*_ and *G*_*p*_ are affected by only input and output data **x** and **y**. The commutative loss (Equation 10) increased (Figure 3A), because commutativity between *f*_1_ and *f*_2_ is not generally constrained without negative feedback control. In contrast, when feedback gain of the commutativity is 1, the synaptic weights are affected by the commutative equation between *f*_1_ and *f*_2_ and the feedback error converged to 0 (Figure 3A). The feedback error between two conditions (gain=0 or gain=1) is significantly different regardless of the same initial synaptic weights inside all neural network modules and inputs (Figure 3B, Mann–Whitney *U* = 10,000, *n* = 100, *p* < 2.5e-4, two-tailed). This result indicates that the feedback error at the macro level can change the synaptic weights inside macro-level functions at the micro level and serves as a concrete working example of the concept of whole-to-parts causation.

## 4 Discussions

We propose a whole-to-parts causation mechanism that is a inter-level feedback control of the micro level synaptic weights using the macro level algebraic structural feedback error. In experiment, we observed that changing feedback gain affected the observed supervenient feedback error. This result shows that the supervenient feedback error at the macro level can change the synaptic weights inside macro-level functions at the micro level.

Whole-to-parts causation was considered impossible because of the “overdetermination” problem. However, conventional debates [i.e., Kim's exclusion argument (Kim, 2000)] did not address inter-level negative feedback control. This type of control consists of two events: observing feedback error at the macro level and controlling to reduce it at the micro level. Observation occurs before control. Furthermore, when the macro-level equation that determine the feedback error can be modified independently of changes at the micro level, the macro-level feedback error acquire new causal power to change micro-level activity through inter-level negative feedback. This suggests that this mechanism overcomes the “overdetermination” problem. To define a macro-level equation with new causal power through inter-level negative feedback control, we assume that the macro-level is a set of functions that supervene on physical states of neurons and synapses. In our formulation, the equations are limited to the axioms that define the algebraic structure of the set of functions, because these axioms are well-defined and independent of external causes. The conditions for realizing whole-to-parts causation can be summarized as follows: (1) inter-level negative feedback control, (2) the system consists of a set of mathematical functions that are regulated by the control, and (3) inter-level negative feedback control is used to satisfy the axioms that define the algebraic structure of the set of mathematical functions.

Algebraic structure cannot be described at the micro level. This situation is similar to the contextual emergence (Atmanspacher and Bishop, 2007). The example of contextual emergence is that the macro temperature cannot be described mathematically from the micro thermodynamics alone. New macro-level context is needed to describe the temperature. The context is similar to our superveneint feedback error at the macro level. However contextual emergence differs from our model in that, in contextual emergence, macro-level context cannot change the micro, they cannot change their mechanism through inter-level negative feedback control. Neurons, however, are systems that can change their synaptic weights through negative feedback control. In our formulation, the macro-level supervenient feedback error that cannot be described at the micro level can change the synaptic weights. Thus, contextual emergence is not related to whole-to-parts causation.

Whole-to-parts causation is different from the causation from the higher level to the lower level in the functional hierarchy. Since the higher level and the lower level do not share the same physical entities, the macro-to-micro causation is not whole-to-parts causation. To our knowledge, the examples in Ellis's “Downward Causation” (Ellis, 2009, 2019) are not examples of whole-to-parts causation we proposed.

Conventional models of emergent downward causation (Rosas et al., 2020) assume that the micro-mechanisms are fixed, and they analyze the state-to-state transition of the fixed micro-mechanism and inter-level negative feedback control is not addressed. In the “macro beats micro” model (Hoel et al., 2013; Rosas et al., 2020), they assume a map from a group of the micro states to the (emergent) macro states, which means that the macro states are assumed to be a summary or coarse-grained state of the micro states. This means that the emergent hierarchy they assumed is not structural hierarchy but functional hierarchy. Furthermore, a modular structure to define structural hierarchy was not assumed in the conventional models (Hoel et al., 2013; Rosas et al., 2020). These are different from our whole–parts relationship, and such differences are essential to explain whole-to-parts causation. In addition, contemporary causal emergence works have aimed to analyze causal structures from an information-theoretic perspective, but our approach differs significantly in that we construct models of causal mechanisms. According to (Pearl 2000), data-driven causal analysis is impossible. (Dewhurst 2021) claims that causal emergence is not causation; therefore, we are more interested in elucidating causal mechanisms than in analyzing causal structures from an information-theoretic perspective. In biology, several debates exists on inter-level causation and macro-to-micro causation, however, current literature on downward (Hoel et al., 2013; Mossio et al., 2013; Noble et al., 2019; Rosas et al., 2020), reticulate (Gershenson et al., 2020) causation did not address whole-to-parts causation. Thus, a model of whole-to-parts causation mechanism has yet to be proposed.

In whole–parts relationship, physical entities must be shared by the whole and its parts. Neural network modules at the macro level and their component neurons and synapses at the micro level satisfy such a relation. In our working model, the higher-level in the hierarchy is not a quantity that characterizes the state of the system like macro-to-micro causation (Ellis, 2009) or coarse-grained state derived from the micro states (e.g., average of the micro states) (Rosas et al., 2020), but macro-level functions that are composed of neurons and synapses. Furthermore, the algebraic structural feedback error is determined at the macro level independent of external macro cause. The external macro like an externally given reference value does not supervene on the micro. Therefore, the reference is not suitable for the whole-to-parts causation. In algebraic structure control, equations described by the macro-level functions are used to control the micro synaptic weights.

The proposed formulation applies only to the brain or brain-like systems and is not generalizable to other objects because we assume that the micro-element is a negative feedback control system, not a molecule or a physical state. In a dynamical system, the focus is often on the transition from state to state, and negative feedback control is not addressed because it is believed that negative feedback control requires a reference signal from outside the system. Additionally, an external reference signal is not suitable for modeling autonomous systems such as biological organisms and conscious brains. In the self-organization system, the interaction between particles at the same hierarchical level is the focus and the interaction between the different hierarchical levels in modular structure has not been addressed. In this paper, we proposed a inter-level negative feedback control system using supervenient feedback error at the macro level without the reference signal from outside the system.

Although our formulation of whole-to-parts causation lacks supporting evidence in the living brain, there is also a lack of evidence that would refute the formulation. Whole-to-parts causation has been considered mysterious due to the “overdetermination” problem. Rejecting whole-to-parts causation supports reductionism. However, our model of whole-to-parts causation avoids the “overdetermination” problem. The macro-level equation for determining feedback error is independent of micro-level events and can change the behavior of micro components via negative feedback control. This type of inter-level relationship has never been addressed in the literature on “overdetermination”. Therefore, we can accept the possibility that the brain uses a similar whole-to-parts causation mechanism. We believe that some kind of macro-determinism by whole-to-parts causation mechanism is necessary to explain volitional movements.

In this article, we present the first working model of whole-to-parts causation. We expect this research to contribute to a future understanding of higher-order law, psychological and mathematical laws independent from lower-level neuro-physiological laws, and to the search for evidence of whole-to-parts causation in living organisms.

## Data Availability

The original contributions presented in the study are included in the article/supplementary material, further inquiries can be directed to the corresponding author.

## Footnotes

1 Whole-to-parts causation is related with the nested causation (Mossio et al., 2013) and the individualizing causation (Gontier, 2023).

2 Current literature on inter-level causation often distinguishes between a causation as discussed in the literature and whole-to-parts causation.

3 In this article, “supervenience” has the same meaning as micro-determination and micro-dependence. A change in a supervenient entity cannot occur without a change in the subvenient entities. This relationship is not causal.
